# Causality and exposome

**DOI:** 10.1007/s10654-025-01328-4

**Published:** 2025-11-28

**Authors:** Paolo Vineis

**Affiliations:** School of Public Health, Imperial College London, London, United Kingdom

There was a time in the early 20th century when, thanks to the developments in physics, the idea of cause looked useless. The theories of Ernst Mack influenced Einstein, philosophers like Bertrand Russell and even a writer like Robert Musil, who had written his doctoral thesis on the Austrian physicist. Bertrand Russell, in particular, stated that “All philosophers imagine that causation is one of the fundamental axioms of science, yet oddly enough, in advanced sciences, the word ‘cause’ never occurs … The law of causality, I believe, is a relic of bygone age, surviving, like the monarchy, only because it is erroneously supposed to do no harm ...” [1]. This point of view has never been really taken seriously in medicine. We need the concept of cause, for very practical reasons, to answer to questions like: what is the cause of poliomyelitis? Is tobacco a cause of lung cancer? Is a vaccine effective? The difficulties of disentangling the multiple connections between variables in observational science (particularly in biology, where everything is variable and dynamic) led to the centrality of experiments and particularly the Randomized Trial as the “gold standard”, an attitude that can be found e.g. in Miettinen’s [2] and Rothman’s [3] classical approaches to causality, i.e. a critical analysis of bias derived from the basic principles of randomization. Things have then evolved for example with the introduction of biological mechanisms in the reasoning, to confer plausibility to empirical observations [4].

However, it seems that the new wave of computation in science (and beyond), based on (deep) machine learning, and generically AI-derived methods, including Large Language Models, brings us back to Russell’s viewpoint. A popular article by Chris Anderson in Wired in 2008 stated: “Petabytes allow us to say: “Correlation is enough.” We can stop looking for models. We can analyze the data without hypotheses about what it might show. We can throw the numbers into the biggest computing clusters the world has ever seen and let statistical algorithms find patterns where science cannot” [5]. This approach has in fact become widespread with “agnostic” searches promoted by “exposome” research. Needless to say, there is much confusion in this enthusiastic view, and the Commentary by Ponzano and colleagues in this issue of the journal clarifies the problem [6]. As the authors say, “while discussions on interpretability have largely focused on statistical inference, causal considerations and the practical applicability of findings to inform the design of tangible interventions have received less attention”. The paper is important because it stresses some of the limitations of current statistical methods applied to big data in epidemiology, and in particular the exposome research: machine learning is a “black box”, i.e. it is very difficult if not impossible to disentangle the role played by single variables (causes, confounders or effect modifiers) in the absence of a logical approach that is not purely statistical, but is based on prior knowledge, assumptions, models and theories. The authors propose that interpretability needs to be subdivided into (at least) three components: (a) statistical inference; (b) causal inference; and (c) actionability (“referring to the degree to which the outputs can be translated into potential public health messages, interventions, and recommendations”). In exposome research, instead, it is a common practice to incorporate all available and measurable components of the exposome into a single machine learning model, without sufficient consideration of the potential causal relationships among these variables. In fact, the idea of a contrast between the RCT-based tradition, founded on the isolation of single causes, and the enthusiasm for radical data-driven empiricism of AI represents an oversimplification. There are other intermediate traditions in contemporary epidemiology: contributions from philosophy of science (Salmon [7], Schaffner [8]); new statistical approaches based on graphs (Directed Acyclic Graphs), structural equation models, mediation analysis, Ordinary Differential Equations, Complex Systems Dynamic methods [9], etc; and models of causality based on molecular pathways. The latter is probably my favourite approach, based for example on the “Salmon-Dowe view” [10], where causal processes are conceptualized as *world lines of objects (*the sequence of spacetime events corresponding to the history of an object). An airplane flying in the sky is a world line, but so is its shadow on the ground. An important question concerns how to discriminate between world lines, or processes, that are causal and those that are not. In the Salmon- Dowe approach, causal processes are capable of transmitting conserved quantities, such as mass-energy, linear momentum, or charge. So, two airplanes that move in the sky and that eventually collide are causal processes, as either process is modified after the interaction. This is however not true of the corresponding shadows on the ground, that are not modified when they intersect. Illari and Russo [11] have suggested to conceptualize the detection and tracing of signals in a world line of objects in terms of *information transmission*. They say that (biological) mechanisms are *information channels*: in fact, information transmission is the most general concept that applies to molecules (e.g. when a chemical reacts with a biological receptor) but also to psycho-social interactions. Transmission of information always involves a stressor and a receptor and can be represented not only with mathematical quantities but also with graphical representations (arrows and nodes) and mechanistic reasoning [12].

The different traditions of thought I referred to above find a unifying vision in Judea Pearl’s theory that considers statistics (associations) as the ground zero, incapable of directly addressing causal questions. Pearls’ approach [13, 14] is similar to the proposal by Ponzano and others, since it distinguishes between three layers of reasoning: association (“seeing”), intervention (“doing”) and counterfactuals (“imagining”). Only the use of tools related to all three layers allows causal inference, and such tools have been developed by Pearl and others in the form of arrows, nodes, intermediary variables, “do-operators”, “back-door criterion”, collider bias, and other concepts that have helped clarify complex problems in causality in epidemiology [15]. Also graphical representations enriched with dynamic path analysis are proposed to facilitate the understanding of causal relationships [16]. Other important contributions are worth mentioning, like the “potential outcome” concept introduced by Rubin [17]. This framework considers two potential outcomes for each individual, one that would result from receiving the treatment/exposure and one from not receiving it. The approach allows better quantification of causal effects and treatment of confounding and of complex situations with time-varying treatments.

There are also parts of the essay by Ponzano et al. I disagree with, in particular when the authors say “Factors such as SES or race are usually vague social constructs encompassing complex historical, societal, demographic, and behavioral factors and that are generally non-modifiable. A first step is to unpack the meaning of these variables to identify upstream, downstream, or proximal factors that could offer specific routes for intervention”. Race itself is not actionable, but structural racism is; and SES is vague simply because we epidemiologists tend to use simplified measures of socio-economic position, while social sciences are much more sophisticated. SES is definitely actionable if it is decomposed as suggested by the authors in its different components and pathways. Even worse, epidemiologists have had the tendency to use SES as a confounder, instead of looking at its overarching role in causal models. In fact, the decision whether certain distal determinants (racism, socioeconomic status) should be included in a model only as confounders/effect modifiers or as true independent variables (and therefore, hypothetically, actionable) has a strong value-laden connotation that influences the interpretation of results and that should be made explicit, particularly in complex models such as those related to the exposome. We should never forget that biology and biographies cannot be easily separated, and mixed *biosocial mechanisms* operate in the real world [18].

The paper by Ponzano and colleagues is welcome to start a discussion in epidemiology that goes beyond simplifications like the use of Mendelian Randomization as the only approach that allows causal inferences, and addresses the conundrums we encounter with data-driven exposome research. This is well represented by the example proposed by the authors, a study aimed at investigating the determinants of cardiovascular diseases using an exposome-wide approach, i.e. obtaining information on hundreds of potential risk factors (I have modified the example by substituting PM2.5 with ultra-processed food). In their example machine learning is used to screen for potential risk factors and ranks obesity as the most important predictor, followed by intake of ultra-processed food (UPF), and socio-economic status (SES). Based on the results, one would conclude that BMI is the main cause of CVD but could also counter argue that BMI lies in the pathway between UPF intake and CVD risk. Also, the inclusion of SES in the model is problematic because this construct both determines UPF consumption and is a predictor of obesity, i.e., it plays a more complex role in the causal model. A correct interpretation requires that a biological model of the impact of UPF and obesity on CVD is put forward, and assumptions are made on the role of different variables including SES, via e.g. a graphical approach.
